# Effects of deferoxamine on blood-brain barrier disruption after subarachnoid hemorrhage

**DOI:** 10.1371/journal.pone.0172784

**Published:** 2017-03-01

**Authors:** Yanjiang Li, Heng Yang, Wei Ni, Yuxiang Gu

**Affiliations:** Department of Neurosurgery, Huashan Hospital, Fudan University, Shanghai, PR China; Hungarian Academy of Sciences, HUNGARY

## Abstract

Blood brain barrier (BBB) disruption is a key mechanism of subarachnoid hemorrhage (SAH)-induced brain injury. This study examined the mechanism of iron-induced BBB disruption after SAH and investigated the potential therapeutic effect of iron chelation on SAH. Male adult Sprague-Dawley rats had an endovascular perforation of left internal carotid artery bifurcation or sham operation. The rats were treated with deferoxamine (DFX) or vehicle (100mg/kg) for a maximum of 7 days. Brain edema, BBB leakage, behavioral and cognitive impairment were examined. In SAH rat, the peak time of brain edema and BBB impairment in the cortex was at day 3 after SAH. SAH resulted in a significant increase in ferritin expression in the cortex. The ferritin positive cells were colocalized with endothelial cells, pericytes, astrocytes, microglia and neurons. Compared with vehicle, DFX caused less ferritin upregulation, brain water content, BBB impairment, behavioral and cognitive deficits in SAH rats. The results suggest iron overload could be a therapeutic target for SAH induced BBB damage.

## Introduction

Subarachnoid hemorrhage (SAH) is a devastating stroke subtype associated with high morbidity and mortality as a result of multifactorial process[1]. During the last decades, intensified research provided an improved understanding on pathophysiology of SAH, although therapeutic options remain limited[2, 3]. Acute brain edema is recognized as a predominant cause of poor clinical outcome after SAH and is primary attributed to blood-brain barrier disruption after ICH[4]. The endothelial cell contraction and disassembly of tight junctions caused increased vascular permeability and consequent formation of brain edema[5], As yet, it has been clearly elucidated that BBB disruption is a key mechanism of SAH-induced brain injury, however, there is no effective treatment available against brain edema and BBB disruption.

Iron has a major role in SAH-induced brain injury. The breakdown of hemoglobin during blood resolution results in iron overload in acute phase of SAH and causes oxidative injury leading to neuronal cell death. Deferoxamine (DFX), an iron chelator, effectively reduced oxidative stress and neuronal death[6]. Meanwhile, iron toxicity is correlated with BBB damage. Iron overload and mitochondrial free radical production were evident in the microvessel endothelium and resulted in endothelial cell damage in rats after transient forebrain ischemia. DFX attenuated iron accumulation and prevented BBB opening[7].

Thus, we hypothesized that iron overload caused acute BBB disruption after SAH, resulting in consequent brain edema and neurologic deficits. Therefore, in the present study, we examined the mechanism of iron-induced BBB disruption after SAH and investigated the potential therapeutic effect of DFX on SAH.

## Materials and methods

### Animal preparation and intracerebral injection

All animal procedures were approved by the University Committee on Use and Care of Animals, Fudan University. A total of 296 male Sprague-Dawley rats (SLAC Laboratories) at age of 3–5 months were used. SAH induction was performed using an endovascular perforation technique as previously described[6]. General anesthesia was initially induced by inhalation of 5% isoflurane. After intubation and initiation of mechanical ventilation, anesthesia was maintained with 2.5 to 3% isoflurane. Blood was obtained from the catheter for analysis of pH, PaO2, PaCO2, hematocrit, and blood glucose. Core body temperature was kept at 36.0 ± 1.0°C with a feedback-controlled heating pad. In a supine position, a midline incision was made to expose the left common carotid artery (CCA) under a surgical microscope. After the left external carotid artery (ECA) was isolated, it was transected distally, and reflected caudally in line with the internal carotid artery (ICA). Thereafter, a 3–0 nylon monofilament suture with heat blunted-tip, was introduced into the left ICA through the ECA stump. After the resistance was encountered, the filament was carefully advanced to perforate the ICA bifurcation and was immediately withdrawn to the proximal ECA, allowing reperfusion and producing hemorrhage. Common carotid artery was temporarily occluded for 2 minutes to limit the hemorrhage volume. Sham operated animals underwent the identical surgical procedure, without insertion of suture. After recovery, rats were housed in an air-conditioned room at 20°C, with *ad libitum* to food and water. The husbandry staffs and veterinarians provided guidance for the animal care including daily observation (every 12 hours per day) and neurological scoring. Neurological scores were evaluated at day 1, 3, 7 and 28 after SAH using a previous published behavior and activity scoring system (Table 1)[8]. The dead rats which were calculated in the mortality included those which died on their own beyond the observation and those which were euthanized because of bad condition. The following conditions are the criteria for euthanasia: 1. behavior and activity score >5; complete anorexia for 24 hours; inability to obtain feed or water; infection (non-healing wounds, organ infection); dyspnea and seizure attack.

**Table 1:** Behavior and activity scores.

Category	behavior	Score
Appetite	Finished meal	0
	Left meal unfinished	1
	Scarcely ate	2
Activity	Walk and reach at least three corners of the cage	0
	Walk with some stimulations	1
	Almost always lying down	2
Deficits	No deficits	0
	Unstable walk	1
	Impossible to walk	2

### Experimental groups

The animals were divided into 4 groups: sham, SAH, SAH+vehicle and SAH+DFX (100mg/kg) group. DFX was administered intraperitoneally 2 and 6 hours after hemorrhage followed by every 12 hours for a maximum of 7 days. The same time course and dosage of saline was administered in the SAH+vehicle group. Afterwards, rats underwent behavioral testing and were euthanized at day 1, 3, 7 and 28 for brain water content calculation, immunohistochemistry or western blot assays.

The study was performed in three parts. Part 1 measured the brain water content, Evan’s blue extravasation and ultrastructural abnormalities at day 1, 3 and 7 after SAH to evaluate the time dependent changes in brain edema and BBB disruption (n = 4 per time point and group). Part 2 investigated the role of iron in SAH-induced BBB disruption at day 1, 3 and 7 by brain water content (n = 4, per time point and group), Evan’s blue extravasation (n = 4, per time point and group), transmission electron microscopy (n = 4, per time point and group), immunohistochemistry (n = 4, per time point and group) and western blot analysis (n = 3, per time point and group). Part 3 compared the acute (n = 61, per group at day 1; n = 42, per group at day 3; n = 23, per group at day 7) and long term (n = 4, per group at day 28) neurological function after SAH in each group to determine the effect of iron chelation on SAH-induced neurologic impairment.

### Brain water content

The determination of brain water content was performed as described in the previous literature[9]. Animals were euthanized with intraperitoneal pentobarbital 60mg/kg injection and decapitated immediately. After the brains were removed, a 3-mm thick coronal brain slice was cut 4mm from the frontal pole and divided into two hemispheres along the midline. Afterwards, each hemisphere was dissected into cortex and basal ganglia. The cerebellum served as controls. The samples from each brain were immediately weighed on an electric analytical balance (model JA2003; Liangping Instrument, Shanghai, CHN) to obtain the wet weight. Then, the brain samples were dried in a gravity oven (model DHG-9123A, Yiheng Technology, Shanghai, CHN) at 100°C for 48 hours to obtain the dry weight. The brain water content was calculated as follows: (wet weight—dry weight)/wet weight.

### Electron microscopy

Electron microscopy was performed as previously described[10]. Rats were euthanized with pentobarbital and subjected to transcardiac perfusion with 4% paraformaldehyde and 2.5% glutaraldehyde in 0.1 mol/L Sorensen’s buffer (pH 7.4). The brains were removed and a 1-mm-thick coronal slice was cut with a blade 4mm from the frontal pole. The cortex and basal ganglia were sampled and immersed in the same fixative overnight at 4°C. Samples were then post-fixed with 1.0% OsO4 and dehydrated in graded ethyl alcohol. After dehydration, samples were infiltrated with propylene oxide, embedded in epon-araldite epoxy resin and sectioned at 60nm. The Ultra-thin sections were then stained with uranyl acetate and Reynold’s lead citrate, and evaluated using a JEOL JEM-1230 transmission electron microscope.

### Evans blue

The integrity of the BBB was investigated by measuring the extravasation of Evans blue. Evans blue dye (2% in saline, 4 mL/kg) was given intravenously at day 1, 3 and 7 after SAH. The animals were perfused with 0.1 mol/L phosphate-buffered saline through the left ventricle to remove the intravascular localized dye until colorless perfusion fluid was obtained from the right atrium. After decapitation, the brain was removed and dissected into left and right hemispheres and each hemisphere was weighed. Brain samples were then placed in 3 mL 50% trichloroacetic acid solution and then homogenized and centrifuged (10 000 rpm for 20 minutes). The supernatant was measured at 610 nm for absorbance using a spectrophotometer (Ultrospec 3; Pharmacia LKB). The tissue content of Evans blue was quantified from a linear standard curve and was expressed as micrograms per gram of brain tissue.

### Immunohistochemistry staining

Rats were euthanized with pentobarbital and perfused with 4% paraformaldehyde in 0.1mM phosphate-buffered saline (pH 7.4). Brains were harvested and kept in 4% paraformaldehyde for 24 hours and immersed in 30% sucrose for 3–4 days in cold room under 4°C. After sinking to the bottoms, the brain was embedded in a mixture of 30% sucrose and optimal cutting temperature compound (Sakura Finetek, Inc., Torrance, USA) at a ratio of 1:2 and 18μm slices were sectioned on a cryostat and then preserved in -80°C freezer. Immunohistochemistry studies were performed as previously described[11]. The primary antibodies were polyclonal rabbit anti-human ferritin IgG (AbD; 1:500 dilution), polyclonal goat anti-occludin IgG (Abcam, 1:500 dilution), polyclonal goat anti-ZO-1 IgG (Millipore, 1:500 dilution), polyclonal goat anti-CD31 IgG (Abcam, 1:500 dilution), polyclonal rabbit anti-VEGF IgG (Abcam; 1:200 dilution), monoclonal rabbit anti-GFAP IgG (Thermofisher; 1:500 dilution), polyclonal rabbit anti-claudin-5 IgG (Millipore.; 1:500 dilution), polyclonal goat anti-alpha smooth muscle Actin (SMA) IgG (Millipore.; 1:200 dilution); polyclonal rabbit anti-iba-1 IgG (millipore.; 1:500 dilution); polyclonal rabbit anti-NeuN IgG (millipore.; 1:500 dilution); polyclonal donkey anti-human ferritin IgG (Abcam; 1:500 dilution). The appropriate Alexa-Flour conjugated antibodies (Invitrogen, Grand Island, NY, USA, 1:500) were used as secondary antibodies. The slides were covered with Fluoroshield with DAPI (Sigma-Aldrich, St. Louis, MO, USA). The double labeling was analyzed using a fluorescence microscope (Olympus, BX51).

### Western blotting

Brain tissue was perfused with 0.1mM phosphate-buffered saline (pH 7.4) after euthanasia and bilateral cortex and basal ganglia were sampled. Then, each sample was immersed in western sample buffer and sonicated. Protein concentration was determined by Bio-Rad protein assay kit, and 50μg protein from each sample were separated by sodium dodecyl sulfate-polyacrylamide gel electrophoresis and transferred to a Hybond-C pure nitrocellulose membrane (Amersham). The primary antibodies were polyclonal goat anti-ZO-1 IgG (Millipore, 1:2000 dilution), polyclonal goat anti-occludin IgG (Abcam, 1:200 dilution) and polyclonal rabbit anti-claudin-5 IgG (Millipore.; 1:500 dilution). Membranes were scanned and the relative densities of bands were analyzed with Image J (version 1.49, NIH). Protein levels were expressed as a fraction of β-actin in the same lane.

### Morris water maze test

The Morris water maze test was performed between 21 and 28 days after SAH. As previous described[12], an 11-cm diameter platform is submerged in 2 quadrants of a pool that is 109 cm in diameter. The hidden platform test assesses the ability of the rats to find the platform without being able to directly see it, and the rats have to either remember where it is relative to external spatial cues or perform a search. The platform was placed 1 cm under the water surface, and the water was made opaque with white, non-toxic tempera paint. Each rat was released from 1 of 4 locations and was allotted 90 seconds to search for the hidden platform. At the end of each trial, the rat was placed on the platform or allowed to remain on the platform for 30 seconds with prominent spatial cues displayed around the room. Because the investigator was also a spatial cue, they always sat in the same location during each trial. Four trials per day for 5 consecutive days were performed with the location of the platform kept constant. Data are expressed as the time (in seconds) or latency to reach the submerged platform on each day. After the last day of the hidden platform test, a single, 60-second probe trial was performed. The platform was removed and each rat was placed in the pool once for 60 seconds, at the same starting location as was used first in hidden platform testing. The time spent in the goal quadrant (where the platform had been located) and the swimming speed were both recorded.

### Statistical analysis

All the data in this study are presented as mean ± SD and analyzed using JMP 12 software (SAS Institute Inc, Cary, UC). Statistical difference among the groups were analyzed by one-way ANOVA with post hoc Bonferroni-Dunn correction for multiple comparisons. P<0.05 was considered statistically significant.

## Results and discussion

### Physiological variables and mortality rate

All physiological variables in all animal groups were recorded before filament insertion. Mean arterial blood pressure, blood pH, PaO2 and PaCO2, hematocrit, and blood glucose were controlled within normal ranges (mean arterial blood pressure, 80–120 mm Hg; blood pH, 7.30–7.50; PaO2, 80–120 mm Hg; PaCO2, 35–45 mm Hg; hematocrit, 35–55%; glucose, 80–130 mg/dL). No sham-operated controlled animals died. The mortality rate in the SAH group and SAH+vehicle group was 28.8%(23 of 80 rats) and 29.1%(25 of 86 rats), respectively. DFX administration lowered the mortality rate to 11.6%(8 of 69 rats, p<0.05). All animals in both SAH and SAH+DFX groups had extensive SAH at 24 hours after operation. The score was 12.9±2.5 and 13.3±2.1 in the SAH groups with or without DFX treatment.

### SAH caused the most severe BBB disruption in cortex at day 3

**Fig 1:**
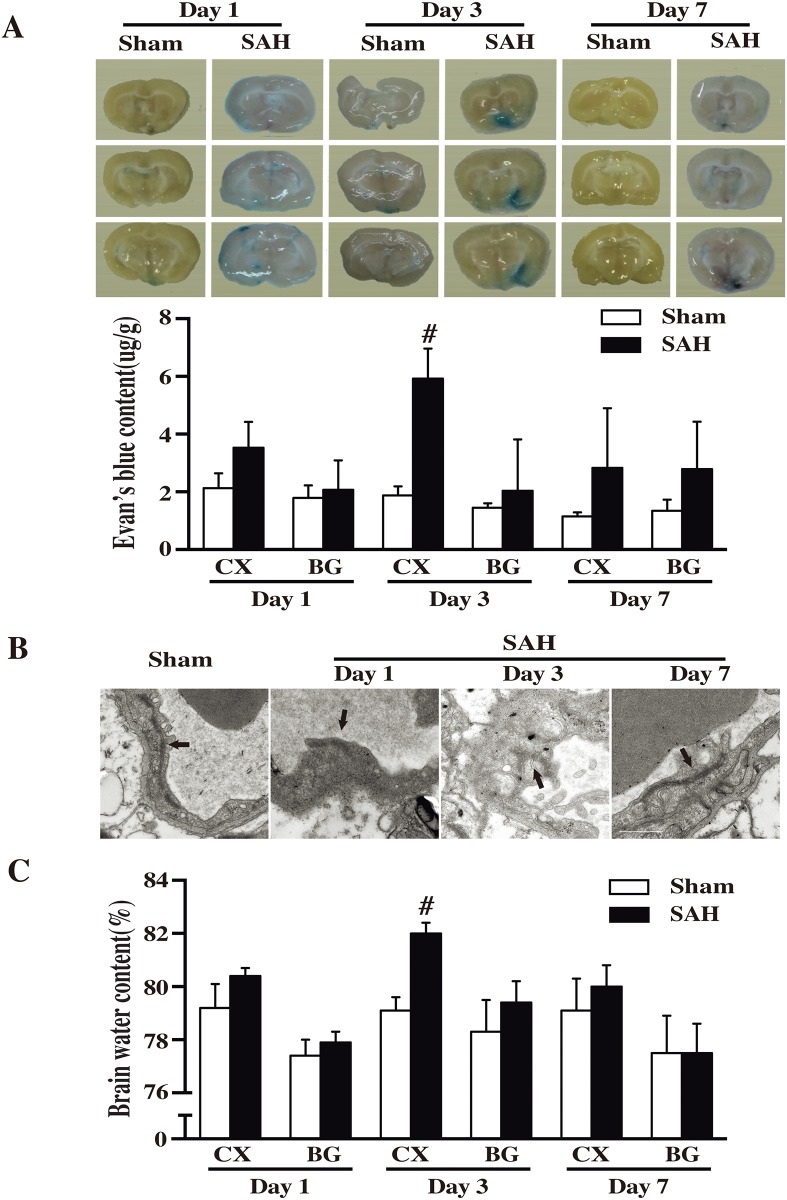
(A) Evan’s blue leakage at day 3 after subarachnoid hemorrhage (SAH) induction or sham. Evan’s blue content in cortex (CX) and basal ganglia (BG) at days 1, 3 and 7 after SAH induction. Values are mean±SD; #p<0.01 versus sham group. (B) Dark arrows showing ultrastructure of tight junction in cortex microvessels after sham operation or SAH at day 1, 3 and 7. Tight junction detachment and basement membrane irregularities were observed at day 3 after SAH. (C) Brain water content in cortex (CX) and basal ganglia (BG) at day 1, 3 and 7 after SAH induction. Values are mean±SD; #p<0.01 versus sham group.

Rats that underwent SAH developed marked Evan’s blue extravasation in cortex, but not in basal ganglia and SAH induced most significant Evan’s blue extravasation in cortex at day 3 (5.92±1.04 versus 1.88±0.31 ug/g; P<0.01; n = 4 for each), but not at day 1 and day 7 (Fig 1A). Electron microscope examination showed ultrastructural abnormalities in cortex microvessels after sham operation and SAH. The tight junction detachment and basement membrane irregularities, indicating BBB disruption, were observed at day 3 after SAH, whereas not at day 1 and day 7 (Fig 1B). In addition, brain water content was increased in the cortex 24 hours after SAH. The peak time of brain water content was also day 3 after SAH (82.04±0.42 versus 79.10±0.51%; P<0.01; n = 4; Fig 1C). These results strongly suggested that SAH causes most severe BBB disruption in cortex at day 3. This injury healed after this peak.

### DFX reduced BBB disruption after SAH

**Fig 2:**
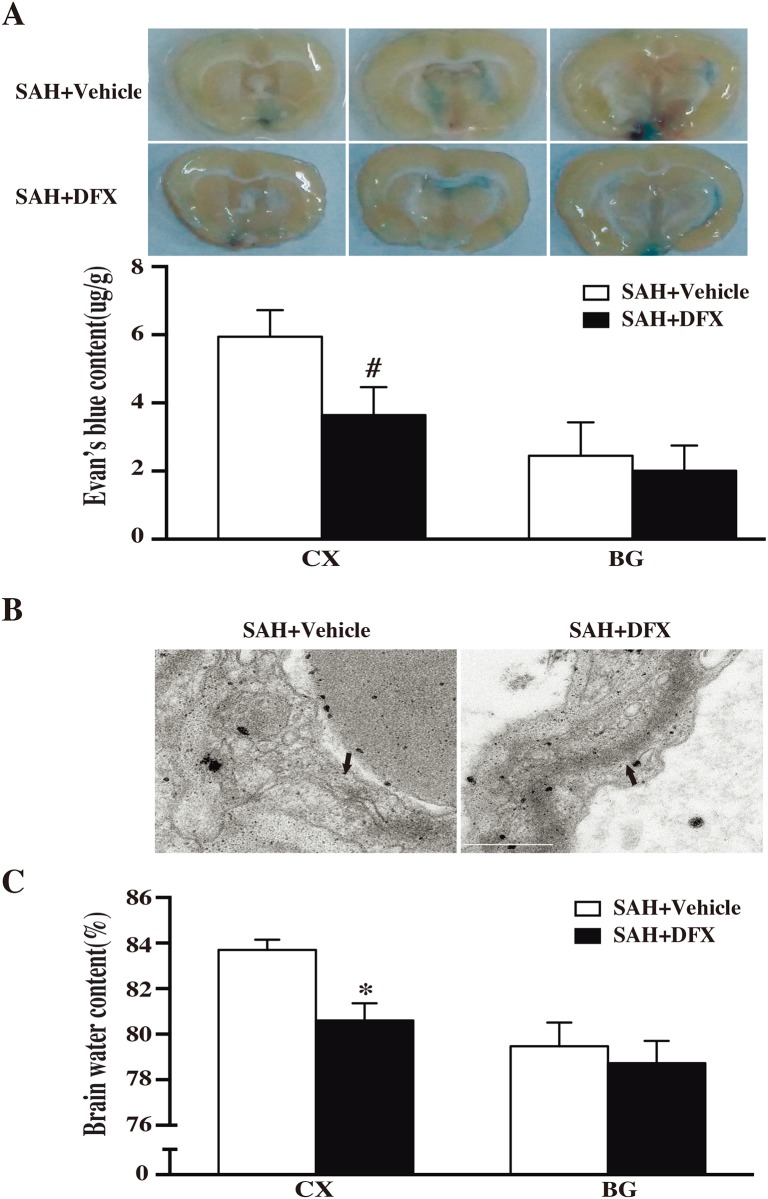
**(A)** Evan’s blue content (CX) in cortex or basal ganglia (BG) at day 3 after subarachnoid hemorrhage (SAH) induction with deferoxamine (DFX) treatment or vehicle. Values are mean±SD; #p<0.01 versus sham group. **(B)** Dark arrows showing ultrastructure of tight junction in cortex microvessels after SAH with DFX or vehicle at day 3. Tight junction detachment and basement membrane irregularities were observed at day 3 after SAH and attenuated by DFX. **(C)** Brain water content in cortex (CX) and basal ganglia (BG) at day 3 SAH induction with vehicle or DFX. Values are mean±SD; #p<0.01 versus sham group.

The effect of DFX treatment was assessed 3 days after induction of SAH. Systemic administration of DFX attenuated Evan’s blue extravasation in cortex at day 3 after SAH (3.64±0.82 versus 5.94±0.78 ug/g; p<0.01; n = 4; Fig 2A). In electron microscope examination at day 3, DFX ameliorated the tight junction detachment and preserved the integrity of the base membrane (Fig 2B). The brain water content in the cortex was also lower in SAH+DFX group than that in SAH+vehicle group (80.6±0.76 versus 83.7±0.45%; p<0.05; n = 4; Fig 2C).

### DFX attenuated degradation of BBB proteins after SAH

**Fig 3:**
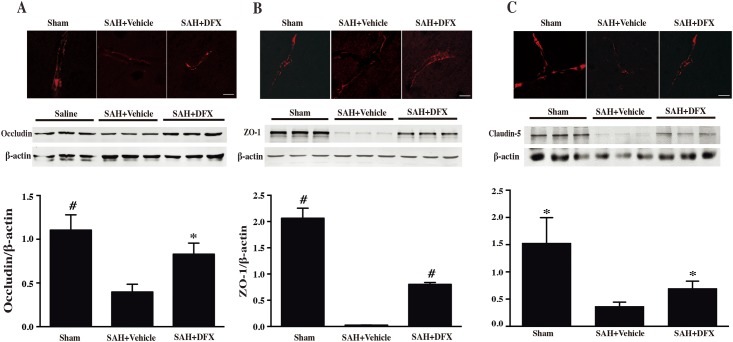
**(A**) Occludin immunoreactivity and protein levels in cortex after sham or subarachnoid hemorrhage induction with deferoxamine (DFX) treatment or vehicle at day 3, scale bar = 20μm. Values are mean ± SD; n = 3 for each group, #p<0.01, *p<0.05 vs. SAH+vehicle group at day 3. **(B)** ZO-1 immunoreactivity and protein levels in cortex after sham or subarachnoid hemorrhage induction with deferoxamine (DFX) treatment or vehicle at day 3, scale bar = 20μm. Values are mean ± SD; n = 3 for each group, #p<0.01 vs. SAH+vehicle group at day 3. **(C)** Claudin-5 immunoreactivity and protein levels in cortex after sham or subarachnoid hemorrhage induction with deferoxamine (DFX) treatment or vehicle at day 3, scale bar = 20μm. Values are mean ± SD; n = 3 for each group, *p<0.05 vs. SAH+vehicle group at day 3.

Occludin is a transmembrane protein that links adjacent endothelial cells and contributes to tight junction stability and barrier function[13]. ZO-1 has been demonstrated to interact with occludin to organize components of the tight junction and link them to the cortical actin cytoskeleton [14]. Claudin-5 is a key component of the tight junction strand, particularly in brain endothelial cells[15]. These proteins are degraded in stroke-induced BBB damage. Given the role of iron in SAH induced brain injury and BBB damage, the protein levels of occluding, ZO-1 and claudin-5 were measured to elucidate whether iron chelation reduced degradation of tight junction proteins (TJPs). Both immunohistochemistry and western blot showed SAH induced significant degradation of occluding, ZO-1 and claudin-5 in the cortex at day 3 after SAH. TJP degradation was effectively prevented by systemic administration of DFX (Fig 3).

### DFX reduced iron overload in endothelial cells in BBB

**Fig 4:**
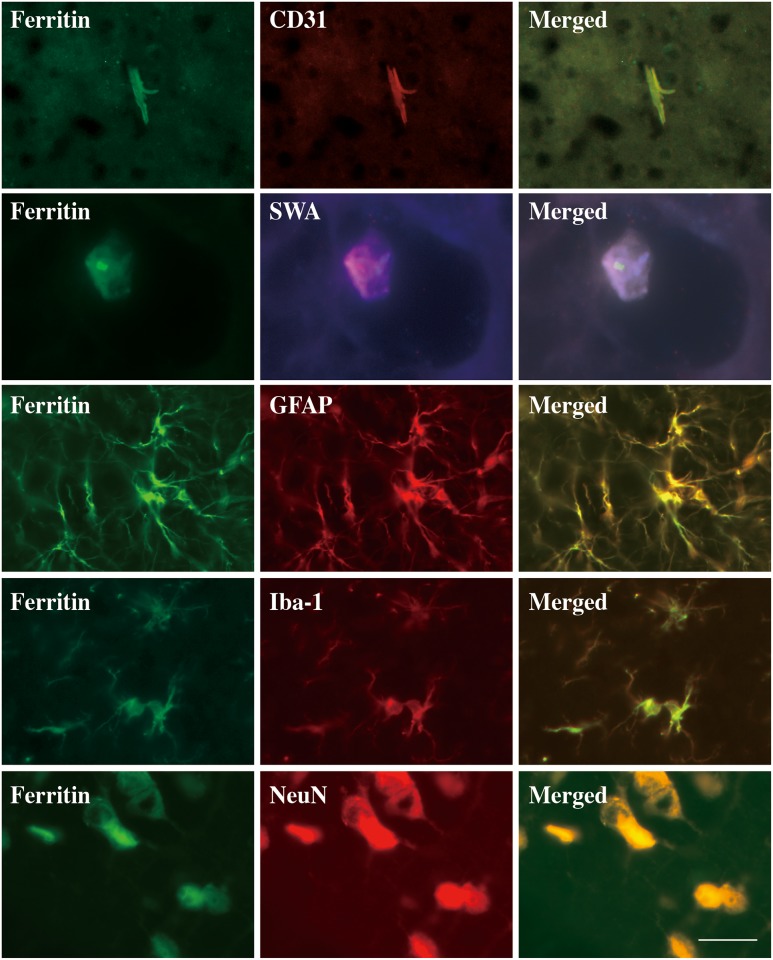
Double labeling of ferritin with CD31(endothelial marker), SWA (a pericyte marker), GFAP (an astrocyte marker), Iba-1 (a microglia marker) and NeuN (a neuron marker)in the cortex at day 3 after subarachnoid hemorrhage. Scale bar = 20 μm.

**Fig 5:**
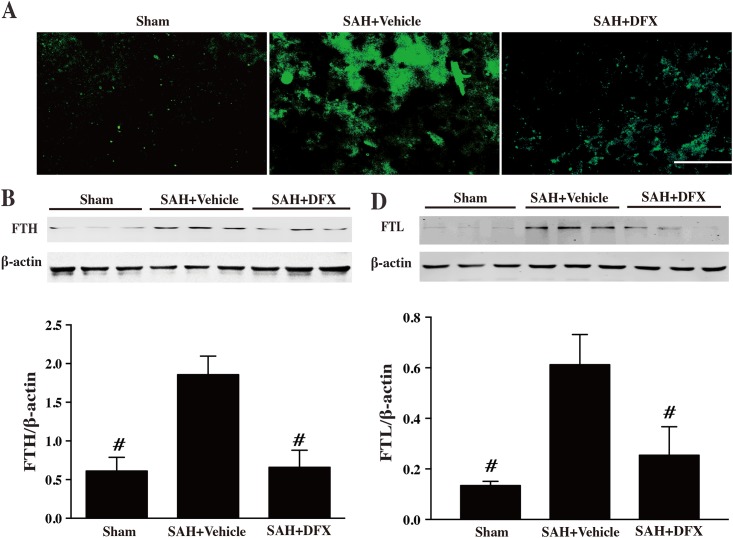
Ferritin immunoreactivity, and the ferritin heavy chain (FTH) and light chain (FTL) protein levels in cortex at day 3 after sham or subarachnoid hemorrhage induction with deferoxamine (DFX) treatment or vehicle, scale bar = 20μm. Values are mean ± SD; n = 3 for each group, #p<0.01 vs. SAH+vehicle group at day 3.

Ferritin, an iron storage protein, was evaluated at day 3 after SAH. To figure out the relationship between iron overload and BBB, Immunofluorenscent double labeling was performed at day 3 after SAH. The result showed that ferritin immunoreactivity colocalized with CD31 (an endothelial cell marker), SWA (a pericyte marker), GFAP (an astrocyte marker), Iba-1 (a microglia marker) and NeuN (a neuron marker), indicating iron overload occurred in endothelial cells, pericyotes and astrocytes, which are the major components of BBB (Fig 4). SAH caused ferritin upregulation at day 3 in the cortex. At this time point, both of the ferritin-positive cells and ferritin protein levels were increased. Systemic DFX administration significantly reduced ferritin expression at day 3 in the cortex (Fig 5).

### DFX improved neurologic behavior and cognitive deficits after experimental SAH

**Fig 6:**
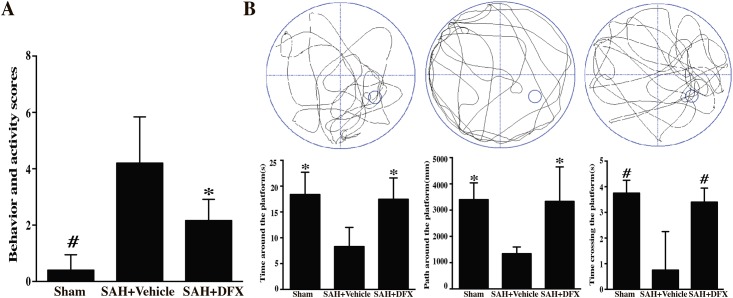
**(A**) Behavioral and activity scores at days 3 after sham or subarachnoid hemorrhage induction with deferoxamine (DFX) treatment or vehicle. Values are mean ± SD; #p<0.01, *p<0.05 vs. SAH+vehicle group at day 3. **(B**) Effect of deferoxamine on cognitive deficits at day 28 after sham or subarachnoid hemorrhage induction with deferoxamine (DFX) treatment or vehicle. Values are mean ± SD; #p<0.01, *p<0.05 vs. SAH+vehicle group at day 28.

The rats showed severe neurologic behavior impairment after induction of SAH. The treatment of DFX resulted in less behavioral impairment at day 3. Next, we examined efficacy of DFX against long-term cognitive injury via the Morris water maze test. After SAH, rats in the vehicle group had weaker memory recall and spent less time and distance searching around or across the quadrant where the platform was located before it was hidden. However, rats with DFX treatment spent more time and distance searching in this quadrant. This indicate rats with DFX had an improvement in performance of the spatial memory in comparison with vehicle group (Fig 6).

The major findings of this study are as follows: (1) SAH causes most severe BBB disruption in the cortex at day 3 after episode; (2) Iron overload occurs in BBB; (3) Administration of DFX attenuated BBB disruption and improved behavior and cognitive deficits after SAH. These results suggest that targeting iron overload may be important for BBB protection after SAH.

BBB disruption is a major component of early brain injury after SAH, which could lead to brain edema formation, followed with brain swelling, increased intracranial pressure, neuron death and neurologic deficits[4, 16]. Previous studies indicated that brain edema in acute phase predominantly referred to accumulation of water as a direct result of BBB disruption[17]. Leukocyte migration followed by BBB disruption may also aggravate brain injury[18]. Thus, it is important to access the BBB disruption after SAH.

The BBB is formed by endothelial cells, which are connected through tight junction proteins, pericytes, astrocytes, neurons and the extracellular matrix. Iron, which derived from blood degradation has been implicated in BBB dysfunction after hemorrhagic stroke[18]. In the present SAH model induced by ICA perforation, BBB permeability occurred and reached a peak at day 3 after SAH induction, accompanied with obvious microstructural impairment (tight junction detachment and basement membrane irregularities) and worst brain swelling. Ferritin, an iron storage protein, is upregulated in brain after SAH. Numerous ferritin-positive cells are colocalized with BBB relevant cells, such as endothelial cells, pericytes, astrocytes and neurons. This indicates that cellular iron accumulation might occur in BBB after SAH and contribute to the BBB disruption after SAH.

Many factors are proved to be contributed to BBB disruption after SAH. For example, SAH induced early brain injury within 24 hours may result in prompt decrease in cerebral blood flow, which deceases brain oxygen supply leading to global ischemia and hypoxia[19]. The consequent nitric oxide (NO)/nitric oxide synthase (NOS) pathways and lipid peroxidation are detrimental to endothelial cells owing to NO-induced neurotoxicity[20]. Platelet activation and aggregation activates MMP-9 and MMP-2 which aggravate BBB disruption[21]. Excessive microglial activation also magnifies BBB damage by releasing proinflammatory mediators[22]. However, SAH induced early brain injury is an explosive damage. Its long-term influence on BBB is not clear. Conversely, iron overload produces long-lasting toxicity to brain. Our previous study demonstrated that iron overload is also involved in BBB breakdown in ICH models. Intracerebral iron injection caused severe BBB disruption followed by albumin leakage [9, 23]. After SAH, there is a progressive increase of brain nonheme iron accompanied with a considerable upregulation of iron-handling proteins[6]. The blood in subarachnoid space may be a source of iron which gradually worsens BBB disruption via the persistent iron release into the brain surface. Therefore, the iron toxicity may cause more profound and persistent damage to BBB. Furthermore, systemic administration of DFX, an iron chelator, effectively reduced brain edema and microstructural impairment in BBB at 3 days after SAH, further supporting our hypotheses.

At present, the mechanisms associated with iron-mediated BBB dysfunction remain incompletely clarified. Iron has a detrimental impact on BBB. Iron overload mediates endothelial cell damage and BBB opening after transient forebrain ischemia. This effect is attenuated by DFX[7, 24]. Our study demonstrated that ferritin was upregulated after SAH in the cortex and was colocalized with endothelial cells, pericytes and astrocytes, which composing of BBB. Ferritin, an iron donor stored in brain endothelial cells, caused low oxygen tension, high levels of the superoxide anion, and acidosis[25–27]. Thus, Iron-mediated free radical production may contribute to BBB disruption. Iron-induced brain damage may also result from oxidation. Iron catalyzes Haber-Weiss reaction resulting in the production of the extremely reactive hydroxyl radical[28, 29], which cause endothelial cell damage and activate cell signaling pathways regulating BBB permeability[29, 30].

Although iron overload is considered as an element cause BBB dysfunction, the effect of iron chelator, DFX, on BBB disruption is controversial. Some studies reported a detrimental effect of DFX in the barrier integrity on brain ischemia models[31–33]. It should be noted that the mechanisms of ischemic and hemorrhagic brain injury differ and that neither ischemia nor BBB leakage play an important role on brain injury at 3 days after SAH. Both our previous studies on ICH or current study on SAH showed that iron chelation attenuated brain edema, microstructural BBB disruption and neurological deficits[9, 34]. These results support the theory that iron chelation may be beneficial for patients with hemorrhagic pathologies.

After SAH, the surface of the brain is covered with high concentration of hemoglobin and its degraded products. Thus, we hypothesized that cortical injury plays a major role in the pathogenesis of early brain injury after SAH. In the current study, we found that DFX reduced ICH-induced neurological deficits in rat at day 3 after SAH. The current behavioral test appears to be well suited to models of wide-spreading brain injury after SAH. DFX improved cognitive dysfunction after SAH. Hippocampus plays a crucial role in the cognitive function. This suggested that the BBB permeability in the hippocampus might also be affected after SAH. Iron chelation might reverse cognitive dysfunction by protecting BBB integrity in hippocampus.

## Conclusion

In conclusion, SAH results in iron overload in BBB, leading to acute BBB disruption, brain edema, neurologic deficits and cognitive dysfunction. Iron chelation effectively reduced acute BBB disruption and attenuated consequent brain edema and neurologic impairment. Hence, iron overload could be a therapeutic target for SAH induced BBB damage.

## Supporting information

S1 Fig VEGF immunoreactivity in cortex at day 3 after sham or subarachnoid hemorrhage induction with deferoxamine (DFX) treatment or vehicle, scale bar = 20μm. Values are mean ± SD; n = 3 for each group, #p<0.01, *p<0.05vs. SAH+vehicle group at day 3.
(TIF)

S2 Fig The timepoints at which the animals died. (TIF)

S1 Table Rats who died due to severe SAH. (DOCX)

S2 Table Symptoms prior to deaths. (DOCX)
