# Correction: Effects of deferoxamine on blood-brain barrier disruption after subarachnoid hemorrhage

**DOI:** 10.1371/journal.pone.0337371

**Published:** 2025-11-24

**Authors:** Yanjiang Li, Heng Yang, Wei Ni, Yuxiang Gu

Following the publication of this article [1], concerns were raised regarding the results presented in Fig 3. Specifically, that the Sham panel of Fig 3B and the SAH + DFX panel of Fig 3C appear to overlap.

Corresponding author YG stated that the Sham panel in Fig 3B is incorrect and provided a corrected version of Fig 3 where the Sham panel in Fig 3B has been updated with the correct panel from the original experiments. The available underlying data for the corrected Fig 3 are provided here in S3 File.

Upon further editorial investigation, it was noted that Fig 5C was mislabeled as Fig 5D. An updated version of Fig 5 is provided here with the correct labeling. The available underlying data for Fig 5 are provided here in S4 File.

In addition, the IACUC approval number for the animal studies was omitted from the “Animal preparation and intracerebral injection” section of the Materials and Methods. Corresponding author YG stated that the IACUC approval number for this study is 2015 JS-435.

The underlying quantitative data for all figures in [1] are provided here in S1-S5 Files, except the underlying quantitative data for Figures S1 and S2 which corresponding author YG stated are no longer available.

**Fig 3:**
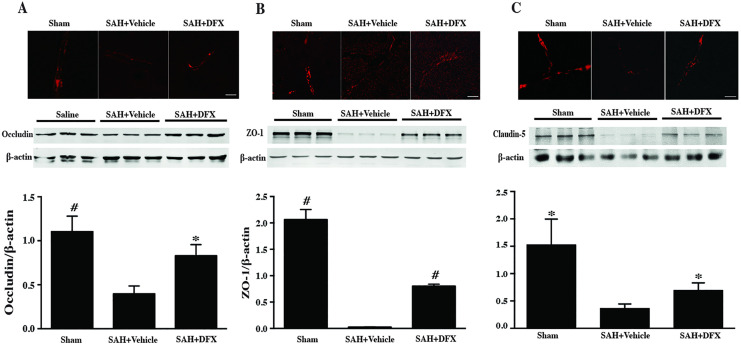
(A) Occludin immunoreactivity and protein levels in cortex after sham or subarachnoid hemorrhage induction with deferoxamine (DFX) treatment or vehicle at day 3, scale bar = 20μm. Values are mean ± SD; n = 3 for each group, #p < 0.01, *p < 0.05 vs. SAH+vehicle group at day 3. **(B)** ZO-1 immunoreactivity and protein levels in cortex after sham or subarachnoid hemorrhage induction with deferoxamine (DFX) treatment or vehicle at day 3, scale bar = 20μm. Values are mean ± SD; n = 3 for each group, #p < 0.01 vs. SAH+vehicle group at day 3. **(C)** Claudin-5 immunoreactivity and protein levels in cortex after sham or subarachnoid hemorrhage induction with deferoxamine (DFX) treatment or vehicle at day 3, scale bar = 20μm. Values are mean ± SD; n = 3 for each group, *p < 0.05 vs. SAH+vehicle group at day 3.

**Fig 5:**
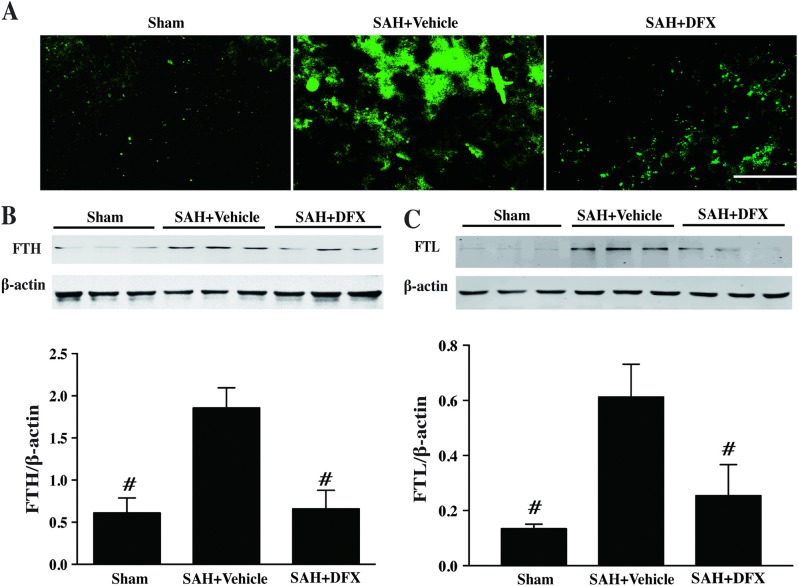
Ferritin immunoreactivity, and the ferritin heavy chain (FTH) and light chain (FTL) protein levels in cortex at day 3 after sham or subarachnoid hemorrhage induction with deferoxamine (DFX) treatment or vehicle, scale bar = 20μm. Values are mean ± SD; n = 3 for each group, #p < 0.01 vs. SAH+vehicle group at day 3.

## Supporting information

S1 File The original underlying quantitative data in support of Fig 1, panels A and C. (ZIP)

S2 File The original underlying quantitative data in support of Fig 2, panels A and C. (ZIP)

S3 File Original underlying data in support of Fig 3. This file includes the quantitative data and microscopy images for panels A-C, the original uncropped Western blot gels for Figs A-B, and the original uncropped western blot for the Claudin-5 panel in Fig 3C. (ZIP)

S4 File Original underlying data in support of Fig 5. This file includes the quantitative data and original, uncropped Western blot gels for panels B and C. (ZIP)

S5 File The original underlying quantitative data in support of Fig 6, panels A and B. (ZIP)
