# Supplementary figures and images for: Effects of deferoxamine on blood-brain barrier disruption after subarachnoid hemorrhage
Source: PLoS One. 2017 Mar 1;12(3):e0172784. doi: 10.1371/journal.pone.0172784 (PMC5332094; doi:10.1371/journal.pone.0172784)

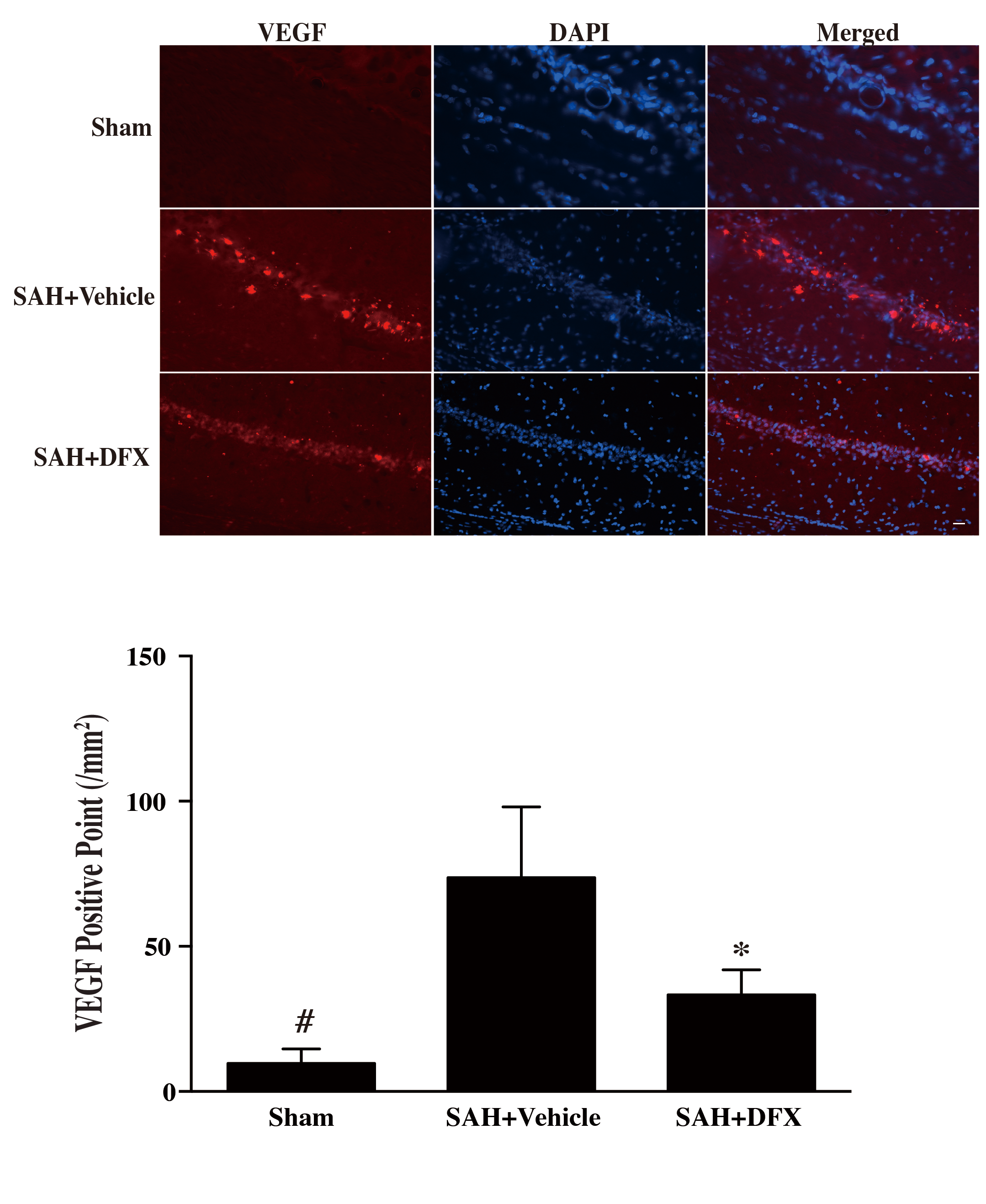

Supplement: S1 Fig — Values are mean ± SD; n = 3 for each group, #p<0.01, *p<0.05vs. SAH+vehicle group at day 3. (TIF) [file pone.0172784.s001.tif]
