# Supplementary material for: Effects of deferoxamine on blood-brain barrier disruption after subarachnoid hemorrhage
Source: PLoS One. 2017 Mar 1;12(3):e0172784. doi: 10.1371/journal.pone.0172784 (PMC5332094; doi:10.1371/journal.pone.0172784)
Supplement: S1 Table — (DOCX) [file pone.0172784.s003.docx]

Supplementary table 1.

| Group | NO. of rats  Died on their own Euthanized | |
| --- | --- | --- |
| SAH | 10 | 10 |
| SAH+Vehicle | 14 | 8 |
| SAH+DFX | 5 | 3 |
