# Supplementary material for: Effects of deferoxamine on blood-brain barrier disruption after subarachnoid hemorrhage
Source: PLoS One. 2017 Mar 1;12(3):e0172784. doi: 10.1371/journal.pone.0172784 (PMC5332094; doi:10.1371/journal.pone.0172784)
Supplement: S2 Table — (DOCX) [file pone.0172784.s004.docx]

Supplementary table 2.

|  | SAH | SAH+Vehicle | SAH+DFX |
| --- | --- | --- | --- |
| **Symptoms prior to euthanasia** |  |  |  |
| *Behavior and activity score >5* | 3 | 2 | 1 |
| *Infection* | 1 |  |  |
| *dyspnea* | 2 | 3 | 1 |
| *Seizure* | 4 | 3 | 1 |
