# Supplementary figures and images for: Correction: Effects of deferoxamine on blood-brain barrier disruption after subarachnoid hemorrhage
Source: PLoS One. 2025 Nov 24;20(11):e0337371. doi: 10.1371/journal.pone.0337371 (PMC12643300; doi:10.1371/journal.pone.0337371)

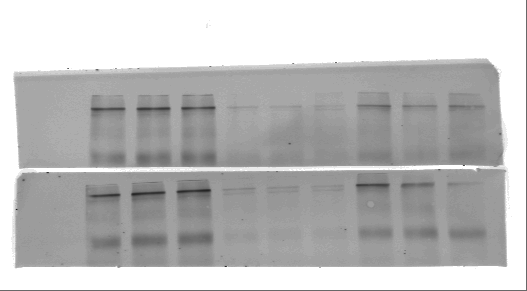

Supplement: S3 File — (ZIP) [file pone.0337371.s003.zip › Figure3c_claudin-5 WB.tif]

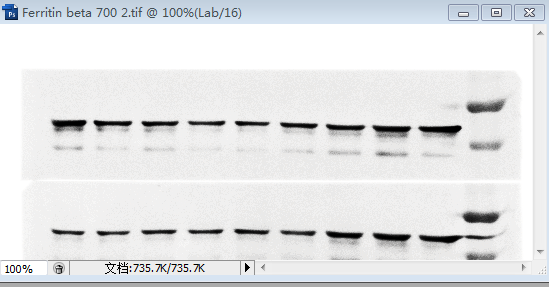

Supplement: S4 File — (ZIP) [file pone.0337371.s004.zip › Figure5b_FTH-beta blot.PNG]

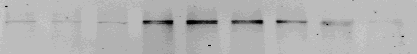

Supplement: S4 File — (ZIP) [file pone.0337371.s004.zip › Figure5c_FTL blot.tif]
